# Paper-Based Electrochemical Devices for the Pharmaceutical Field: State of the Art and Perspectives

**DOI:** 10.3389/fbioe.2020.00339

**Published:** 2020-04-23

**Authors:** Amina Antonacci, Viviana Scognamiglio, Vincenzo Mazzaracchio, Veronica Caratelli, Luca Fiore, Danila Moscone, Fabiana Arduini

**Affiliations:** ^1^Department of Chemical Sciences and Materials Technologies, Institute of Crystallography, National Research Council, Rome, Italy; ^2^Department of Chemical Science and Technologies, Tor Vergata University, Rome, Italy; ^3^SENSE4MED, Rome, Italy

**Keywords:** electrochemical detection, nanomaterials, microfluidics, pharmaceutical sector, paper-based devices

## Abstract

The current international pharmaceutical scenario encompasses several steps in drug production, with complex and extremely long procedures. In the last few decades, scientific research has been trying to offer valid and reliable solutions to replace or support conventional techniques, in order to facilitate drug development procedures. These innovative approaches may have extremely positive effects in the production chain, supplying fast, and cost-effective quality as well as safety tests on active pharmaceutical ingredients (APIs) and their excipients. In this context, the exploitation of electrochemical paper-based analytical devices (ePADs) is still in its infancy, but is particularly promising in the detection of APIs and excipients in tablets, capsules, suppositories, and injections, as well as for pharmacokinetic bioanalysis in real samples.

## Introduction

Drug development represents a long and complex process characterized by several steps, from the recognition of a new molecule with potential therapeutic value to a final product approved for marketing and human health. The synthesis and characterization of such molecules, defined as pharmaceutical compounds, are critical prerequisites for further investigations to create preliminary safety and therapeutic efficacy data (Sinha and Vohora, 2018). To this aim, the pharmaceutical research entails the analytical studies on bulk drug materials, formulations, products, intermediates, impurities, and degradation products, as well as their bioanalyses. These steps are crucial in drug development but represent a weak link in the production chain, due to long-lasting and expensive phases; indeed, drug development takes about 10 years and accounts for two-thirds of the total R&D costs (Rang and Hill, 2013). These main critical concerns are connected to the stringent regulatory authorities in assessing the safety and efficacy of new compounds (e.g., ministry of health, government department including specific regulatory agencies, such as U.S. Food and Drug Administration), hindering a commercial success.

For these reasons, the pharmaceutical industry is strongly interested to improve the entire production chain, not neglecting any technical, investigative, and managerial aspects, to remain profitable and competitive, as in the case of machine learning and artificial intelligence for end-to-end drug discovery and development (Ekins et al., 2019; Mak and Pichika, 2019).

Concerning the methodologies to establish the safety and efficacy of a product pharmacokinetically suitable for human health, the analytical methods conventionally recognized and utilized are titrimetry, spectrometry, chromatography, and capillary electrophoresis. However, all these procedures require technologically advanced equipment and highly skilled personnel (Siddiqui et al., 2017). In this context, a technological revolution is strongly required; therefore, the fervent research of the last decades is not surprising. The exploitation of sensing devices proved to be useful in this sector, being reliable, sensitive, fast, and less expensive than the conventional methodologies. The potentialities of these analytical tools have been recently augmented by novel technologies (e.g., electrochemistry, microfluidics, and nanotechnology), and smart material designed for point-of-use applications (e.g., paper) (Maduraiveeran et al., 2018). In this overall scenario, paper-based analytical devices (PADs) have attracted, in the last years, widespread attention because of their inherent advantages as low cost, easiness to use, free-pump equipment for sample handling and processing, in addition to the advantages of sensitive and selective detection provided by electrochemistry. In addition, microfabrication and microfluidics allowed for the design of novel tools for drug analysis. In particular, microfluidics promises significant improvements not only for its potential to provide reliable and fast devices, but also because it allows to significantly lower production costs, reduce the amount of reagents used during the analysis, and scale down sample volumes to be analyzed (Kang et al., 2008). Lab-on-chip, organs-on-chip, 3D cell culture, and droplet techniques represent recent examples of microfluidic-based systems for basic and applied research in drug screening, drug determination, drug metabolism, and toxicity (Dittrich and Manz, 2006; Xu et al., 2011; Chen et al., 2016).

Moreover, the use of nanomaterials (e.g., metal nanoparticles and carbon nanomaterials) has shown a quantitative influence on the enhancement of electrochemical (bio)sensors and lab-on-a-chip performances, with clearly positive effects on the analyses. Indeed, such nanomaterials have demonstrated benefits for higher electrocatalytic properties and sensing response thanks to their large surface area, defect sites, high electrical conductivity, and good mechanical features (Farka et al., 2017; Piscitelli et al., 2017; Mazzaracchio et al., 2019). Owing to these astonishing features, nanomaterials are currently exploited for *in vivo* and *in vitro* medical applications in the form of robust and tuneable diagnostic and therapeutic platforms (Chen and Chatterjee, 2013).

Finally, in the last decades a strong interest arose on the exploitation of biopolymers in the design of (bio)sensors for pharmaceutical and biomedical sectors, mainly driven by low-cost applications. In particular, paper has shown several advantages (e.g., compatibility with biological samples, environmental sustainability, ease assembling, storage, and transport, and adaptability as support for printing technologies) that make it an ideal substrate in highly engineered diagnostic devices (Yetisen et al., 2013; Meredith et al., 2016; Lee et al., 2018; Noviana et al., 2019). This last requirement represents an important and urgent topic declared by the World Health Organization, which is particularly interested in biomedical research toward the design of sensitive, cost-effective equipment-free diagnostic tools devoted to both developed and developing countries (Urdea et al., 2006).

This review describes the last trends associated with the design of electrochemical paper-based analytical devices (ePADs), as robust, fast, and affordable strategy for drugs analysis during the production process as well as in bioanalyses, highlighting the main advantages of ePADs in comparison with both the conventional methodologies and the bulk electrochemical sensors exploited for the detection of active pharmaceutical ingredients (APIs) and excipients, as well as for pharmacokinetic bioanalysis. In details, in case of comparison with conventional methodologies, ePADs are characterized by the capability to be applied on site by unskilled personnel with cost- effective set-up allowing for a rapid analysis (Table 1). While, in case of comparison with bulk electrodes, ePADs are characterized by lower cost as well as lower volume of sample needed for the analyses combined with the absence of working electrode surface treatment (Table 2).

**TABLE 1:** Main advantages of ePADs in comparison with conventional methodologies for the pharmaceutical sector.

	**Analyte**	**Technique**	**Reagent/substrate modification**	**Volume**	**Matrix**	**Linear range/LOD**	**Comments**	**References**
**e-PAD**	Ascorbic acid	CV	–	100 μL	–	0–5 mM/0.15 mM	Cost-effective and high performant material. Small sample volume. Simple method and fast responsive	Cinti et al. (2018)
	Diclofenac sodium	LSV and DPV	–	10 μL	Tap water	0.10–100 μM/70 nM	Low-cost, disposable and multiplex electroanalytical device for the drug diclofenac	Costa-Rama et al. (2019)
	Dopamine	SWV	SDS	20 μL	Serum	1–100 μM/0.37 μM	Small sample volume. Low-cost. Low detection limits from sample preconcentration	Rattanarat et al. (2012)
	Ketamine	CV	Zeo–GO	20 μL	Urine	0.001–5 nM/mL/0.001 nM/mL	Simple, economical and fast responsive	Narang et al. (2017a)
	Estradiol	DPV	–	10 μL	Serum	0.01–100 ng/mL/10 pg/mL	Low-cost, sensitive, specific, and point-of-care diagnosis of estradiol	Wang Y. et al. (2018)
**Traditional technique**	Ascorbic acid	Titrimetry	2,6-Dichlorophenol indophenol 0.2 g Sodium bicarbonate 0.2 g Phenolphthalein	1000 mL	Commercial liquid diets	166–347 μg/mL	The method is laborious and the standard dye solution is unstable and requires standardization before use. It is subject to interference by other reducing substances.	Hughes (1983)
	Diclofenac sodium	Spectrophotometry	Methylene Blue solution 0.032% w/v (1.04 × 10^–3^M)	30 mL	Tablets	0.6–6.4 μg/mL/0.37 μg/mL	No *in situ* measurements. High sample volume	Botello and Pérez-Caballero, 1994
	Diclofenac sodium	HPLC-UV detection	Methanol–water (60:40, v/v) as the mobile phase	10 μL	Tablets	0.05–0.6 mg/mL	Long analysis times (15 min). Expensive instrument	Kasperek (2008)
	Dopamine	Fluorimetry	Methanol Acetate buffer solution	20 mL	Urine	0.10–3.50 μg/mL	Time consuming and laborious procedures for sample preparation	Sun et al. (2002)
	Dopamine	HPLC-coulometric detection	The mobile phase consisted of 50 mmol/l sodium phosphate, 50 mmol/l sodium acetate, 0.6 mmol/l sodium octanesulfonate, 0.6 mmol/l EDTA and 9 vol.% acetonitrile	1 mL	Rat brain	12–700 ng/g	Long pre-treatment of the chromatography column (10 h)	Bielavskà and Kassa (2000)
	Ketamine	GC-MS	Acidic methanol (containing 1% of HCl) trifluoroacetic anhydride	1 μL	Urine	50–250 ng/mL/2 ng/mL	Time consuming owing to sample derivatization	Lin and Lua (2006)
	Ketamine	LC-MS/MS	Ammonium hydroxide water–methanol (95:5, v/v) 20 mM phosphate buffer (pH 7.4)	10 μL	Urine	4.0–3200 ng/mL/2 ng/mL	Time consuming sample preparation, expensive equipment and skilled person to operate	Lin et al. (2013)
	Estradiol	LC-MS/MS	70% water (solvent A) and 30% methanol/acetonitrile mixture (75/25) (v/v) (solvent B) to 59% solvent A and 41% solvent B from 0.00 to 1.62 min and from 1.62 min on 81% solvent B up to 4.47 min	–	Serum	LOQ 1.3 ng/L (4.8 pmol/L)	No *in situ* measurement. Expensive instruments	Pauwels et al. (2013)

*CV, cyclic voltammetry; DPV, differential pulse voltammetry; EIS, electrochemical impedance spectroscopy; GCE, glassy carbon electrode; GC-MS, gas chromatography–tandem mass spectrometry; HPLC, high performance liquid chromatography; HPLC-MS, high performance liquid chromatography tandem mass spectrometry; HPLC-UV, high performance liquid chromatography tandem ultraviolet spectrophotometry; SWV, square wave voltammetry; LC-MS, liquid chromatography-tandem mass spectrometry.*

**TABLE 2:** Main advantages of ePADs in comparison with and the bulk electrochemical sensors for the pharmaceutical sector.

	**Sensor**	**Analyte**	**Technique**	**Electrode pre-treatment**	**Volume**	**Linear range/LOD**	**Matrix**	**References**
**e-PAD**	CB/inkjet printed electrode	Ascorbic acid	CV	Not needed	100 μL	0–5 mM/0.15 mM	–	Cinti et al. (2018)
	WE: carbon ink, RE and CE: metallic wires	Diclofenac sodium	LSV and DPV	Not needed	10 μL	0.10–100 μM/70 nM	Tap water	Costa-Rama et al. (2019)
	SPE	Dopamine	SWV	Not needed	20 μL	1–100 μM/0.37 μM	Serum	Rattanarat et al. (2012)
	Stencil-printed electrode	Ketamine	CV	Not needed	20 μL	0.001–5 nM/mL/0.001 nM/mL	Urine	Narang et al. (2017a)
	Ab-E/MWCNTs/THI/ AuNPs/SPE	Estradiol	DPV	Not needed	10 μL	0.01–100 ng/mL/10 pg/mL	Serum	Wang Y. et al. (2018)
**Bulk electrodes**	ERGO-GCE	Ascorbic acid	DPV	Cleaning	5–10 mL	0.3–2 mM/0.3 mM	Urine	Yang et al. (2014)
	AuNPs/MWCNT/GCE	Diclofenac sodium	SWV	Al_2_O_3_ polishing, HNO_3_ cleaning and H_2_SO_4_ activation	5–10 mL	0.03–200 μM/20 nM	Urine	Afkhami et al. (2016)
	Graphene/GCE	Dopamine	DPV	Al_2_O_3_ polishing, N_2_ stream drying	5–10 mL	4–100 μM/2.64 μM	–	Kim et al. (2010)
	Ab-K/AuE	Ketamine	EIS	Al_2_O_3_ polishing, HNO_3_, C_2_H_5_OH and ultrapure water cleaning, N_2_ stream drying	5–10 mL	1–100 × 10^–6^ μM/0.41 × 10^–6^ μM	Serum	Chen and Chatterjee (2013)
	Ab-E/Au@Pd NRs/(CoFe_2_O_4_-rGO)/GCE	Estradiol	AMP	Al_2_O_3_ polishing	5–10 mL	0.01–18.0 ng/mL/3.3 pg/mL	River water	Zhang et al. (2016)

*Ab-E, estradiol antibody; Ab-K, ketamine antibody; AMP, amperometry; AuE, gold electrode; AuNPs, gold nanoparticles; Au@Pd NRs, gold core–palladium shell nanorods; CB, carbon black; CoFe_2_O_4_, CoFe_2_O_4_ graphene oxide nanohybrid; CV, cyclic voltammetry; DPV, differential pulse voltammetry; EIS, electrochemical impedance spectroscopy; GCE, glassy carbon electrode; IPE, ink-jet printed electrode; MWCNT, multi-walled carbon nanotubes; RGO, electrochemical reduced graphene oxide; SDS, sodium dodecyl sulfate; SPE, screen-printed electrode; SWV, square wave voltammetry; THI, thionine; Zeo–GO, nanocrystals of zeolite and graphene oxide nanoflakes.*

As also reported in our recent review (Arduini et al., 2017), many electrochemical (bio)sensors have been developed for biomedical applications (Huang X. et al., 2017; Huang Y. et al., 2017; Jiang et al., 2017). This manuscript was inspired by the unequal use of sensors in the pharmaceutical sector, where optical devices are abundant while the electrochemical ones are still in their infancy, despite their potential has been already demonstrated. In particular, the convergence of innovative technologies in the design of advanced tools will be reported to boost the progress in electrochemical sensors devoted to the drug production chain and personalized healthcare.

## Pioneering Pharma-On-Chip: ePADs for the Pharmaceutical Sector

In the last decades, the academic interest toward PADs is considerably growing, to foster the development of sensing platforms for pharmaceutical purposes. Starting from an unbiased analysis of this context, it is noteworthy that the majority of PADs relies on colorimetric detection, despite the intrinsic limitations of this technology as low sensitivity, restricted linear ranges, color saturation problems, and background color due to the matrices (Arduini et al., 2017).

The analytical potential of ePADs has been well-established in literature as customized tools, being able to overcome sensitivity and selectivity drawbacks, as well as to encompass multifarious configurations of sensors (e.g., screen-printed electrodes), bioreceptors (e.g., enzymes, antibodies, artificial molecules), and nanomaterials (e.g., metal nanoparticles, graphene, carbon black) (Adkins et al., 2015).

In a recent review, Noviana et al. (2019) summarized the fascinating and multi-step procedure to obtain ePADs, considering the fabrication of both sensor (e.g., screen-printing, inkjet-printing, stencil-printed, pencil/pen-drawn, and microwire electrodes) and microfluidic pattern (e.g., photolithography, wax printing).

Currently, ePAD applications include clinical diagnosis, environmental monitoring, food analysis, and drug analysis. Moreover, the possibility to obtain a multiplexing sensor which increase the efficiency and accuracy of the analysis has already been demonstrated (Wang H. et al., 2018; Xu et al., 2018). Notwithstanding the potentialities reported above, the global market of paper diagnostics, estimated at USD 5.69 billion in 2017 and projected to reach over USD 9 billion in 2025, does not include any commercialized ePAD to date at our knowledge (Grand View Research, 2019).

In the following section, major milestones achieved with ePADs for *in vitro* and *in vivo* APIs detection were reported, to furnish a current snapshot of the successes obtained, which can become inspiration sources for fine-tuned drug development procedure.

Currently, a large variety of compounds with different origins and chemical properties is routinely used for drugs design, mainly classified into active and inactive pharmaceutical ingredients. These two types of ingredients accomplish different functions, but their union is essential for conservation and effectiveness of the final formulation.

In particular, APIs are defined by WHO as “*Any substance or combination of substances used in a finished pharmaceutical product, intended to furnish pharmacological activity or to otherwise have direct effect in the diagnosis, cure, mitigation, treatment or prevention of disease, or to have direct effect in restoring, correcting or modifying physiological functions in human beings*” (Working document QAS/11.426/Rev.1) (World Health Organization [WHO], 2011). Rigorous and strict standards regulate these compounds, whose compliance is mandatory for every actor in the pharmaceutical production chain (EudraLex, 2011; U.S. Food and Drug administration, 2017). Moreover, a list of APIs sources has been assessed by the WHO and considered acceptable for use in manufacture of finished pharmaceutical products by United Nations (World Health Organization [WHO], 2019). The listed APIs meet WHO norms and standards, as well as the relevant manufacturing sites complying the Good Manufacturing Practices.

Active pharmaceutical ingredients can be mainly identified as drug of synthetic and natural source. The first one includes organic (e.g., acetylsalicylic acid, chloramphenicol) and inorganic synthetic drugs (e.g., aluminum hydroxide, magnesium trisilicate). Natural chemical drugs can be divided in biochemical drugs and plant chemical drugs (Bade et al., 2010; Lahlou, 2013).

On the contrary, inactive pharmaceutical ingredients do not increase or affect the therapeutic action of the active ingredient, but guarantee the dosage, stability, and bioavailability of the active principle (Pifferi and Restani, 2003; Elder et al., 2016). These inert ingredients or excipients (e.g., dyes, preservatives, and flavoring agents) are added during the manufacturing of tablets, capsules, suppositories, and injections, and are approved by FDA. In this context, the US government agency released a guidance for the industries to provide recommendations for use and dosage, as well as to clarify the terminology (U.S. Food and Drug administration, 2019). Excipients can derived from natural sources or synthesized chemically or by other means, such as fermentation (Saluja and Sekhon, 2016).

**FIGURE 1:**
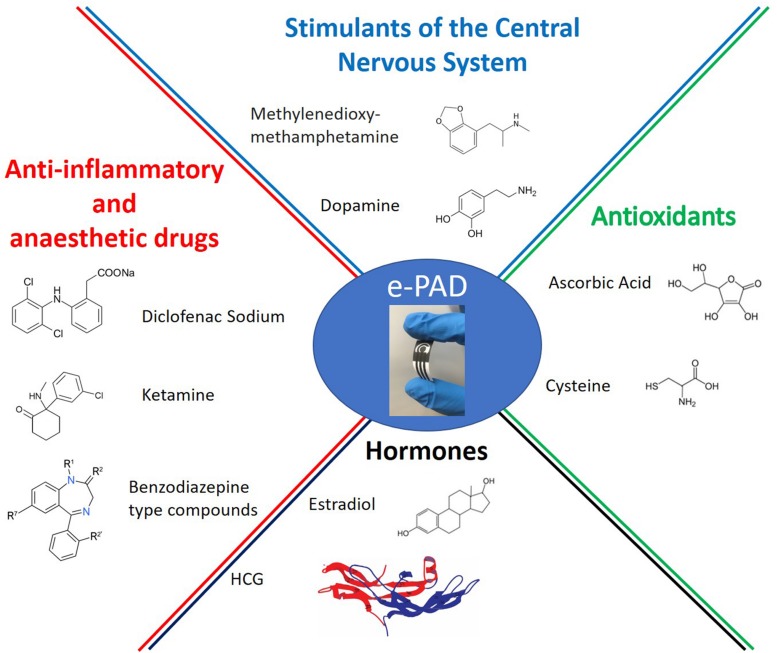
Active pharmaceutical ingredients detected by e-PAD devices.

During the pre-clinical and early-clinical phases, careful analyses on active and inactive pharmaceutical ingredients are performed. These studies concern the compound characterization, the identification of the effective dose and range, as well as the side effects which may occur in tissues. HPLC and UV-Vis spectrophotometry are useful and widely exploited, but they are expensive and time consuming approaches. In this context, handheld and integrated electrochemical sensors, capable of rapid, selective, and sensitive analysis, could be competitive in comparison with conventional techniques for pharmaceutical analysis. Among them, ePADs are promising tools for several pharmaceutical compounds (Figure 1) and the following sections classify paper-based devices in function of the analyte (EudraLex, 2011; Wesoły et al., 2016).

### Antioxidants

Antioxidants are excipients generally exploited to increase physical and chemical stability. Among them, ascorbic acid and cysteine are widely exploited in pharmaceutical industry. In detail, ascorbic acid boosts a substantial therapeutic market as solid tablets and liquid forms, characterized by prolonged storage and with an assured vitamin C content. Ascorbic acid has many functions in the maintenance of various biological activities, including beneficial effects on skin, activity as cofactor in collagen biosynthesis, and antioxidant capacity. In particular, the molecules of ascorbic acid neutralize the free radicals present in the intra and extracellular matrices, avoiding damage to lipid membrane, DNA, and proteins that would be caused by oxidative stress (Maione-Silva et al., 2019). In medicine, its topical application guarantees anti-inflammatory and depigmenting effects (Farris, 2005; Stamford, 2012), while in the pharmaceutical sector ascorbic acid is mainly detected in pharmaceutical products for the quality assessment of, e.g., dietary supplements.

**FIGURE 2:**
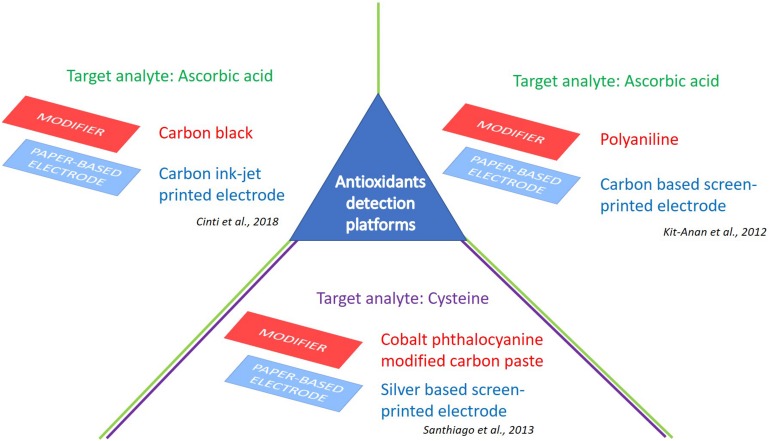
e-PAD platforms for detection of antioxidants. Target analytes: Ascorbic acid (Kit-Anan et al., 2012; Cinti et al., 2018), and cysteine (Santhiago et al., 2013).

A low-cost and disposable ePAD for ascorbic acid pre-screening was realized utilizing inkjet-printed polyaniline (PANI) modified screen-printed carbon electrodes (Kit-Anan et al., 2012) (Figure 2). In detail, this device is constituted of a PANI modified Screen-Printed Carbon Electrode (SPCE) as the working electrode, and two bare SPCEs as reference and counter electrodes, screen-printed on a filter paper. These investigations allowed the authors to present an alternative tool for a real-time detection of ascorbic acid by chronoamperometry, with a sensitivity of 17.7 μA/mM and a detection limit of 30 ± 3 μM in a concentration range of 30–270 μM.

Recently, another example of ePAD tested toward ascorbic acid detection was obtained. This disposable sensor has been modified with a dispersion based on carbon black nanoparticles to increase the electrochemical performances of the printed sensor, allowing for a highly performant nanomodified electrochemical sensor platform (Figure 2). In particular, in the presence of carbon black a decrease of the over-potential for ascorbic acid oxidation was reported (from 0.47 to 0.28 V) compared with bare sensor, as well as a boosted sensitivity (3-times). The proposed electrochemical sensor was able to detect ascorbic acid in a dietary supplement, quantifying 999 ± 130 mg with respect to the 1,000 mg reported on the label (Cinti et al., 2018). The results achieved have shown the reliability of the sensor for controlling the quality and quantity of ascorbic acid present in the dietary supplement, by means of small volumes (i.e., drop) and fast analysis time (i.e., 1 min). This approach highlighted the cost-effectiveness as well as the easiness of use of the proposed paper-based device for API control.

The ePAD technology was also assured for the design of agreeable multi-detection platforms. This is the case of the functional paper-based device constituted by a single-walled carbon nanotube (SWCNT) electrode and a Nafion-modified nitrocellulose membrane. In detail, the device was able to simultaneously detect the presence of both ascorbic acid and active pharmaceutical (acetaminophen) compounds. Using Nafion-modified nitrocellulose membrane in combination with gold nanoparticles and polyglutamic acid for the SWCNT electrode functionalization, a distinguishable acetaminophen oxidation peak was obtained, which was distinct from the ascorbic acid oxidation peak. This allowed for acetaminophen detection in linear range from 50 to 300 μM, which is broader than the standard drug dose range (Lee et al., 2016).

Cysteine, an amino acid usually present in the form of *N*-acetyl-L-cysteine (NAC) in supplements, is another inactive compound widely exploited as pharmacological antioxidant and cytoprotectant. The human body turns NAC into cysteine and then glutathione, a strong antioxidant (Elias et al., 2005). In the pharmaceutical industry, cysteine is used to improve hepatic function and pigmentation, or as antioxidant agent in clinical nutrition and food industry (e.g., in natural fruit juice products).

An interesting ePAD was developed by backfilling small holes made in polyester sheets using a CO_2_ laser etching system. Silver working electrodes were screen-printed on paper in a sandwich two-electrode configuration. The device was tested using linear sweep voltammetry toward cysteine using cobalt phthalocyanine as a redox mediator. The rate constant obtained by chronoamperometry was approximately 10^5^ s^–1^ M^–1^, with a limit of detection of 4.8 μM (Santhiago et al., 2013) (Figure 2).

### Hormones

Several hormones are naturally secreted in microscopic amounts as messengers by the endocrine signaling system. They can be transported by the circulatory system toward distance targets (e.g., tissues, organs), determining an increase or decrease of cellular activities. In specific conditions, a hormonal drug therapy is required to restore the hormonal balance in the patience by either replacing the missing hormone or by inhibiting hormone secretion.

The API estradiol is especially secreted within the follicles of the ovaries, and in the human body is obtained from cholesterol through a series of reactions and intermediates. It is used in menopausal hormone therapy to preclude and treat menopausal symptoms. In fact, its role is pivotal in regulating reproduction in humans; for this reason, several oral contraceptives contain the synthetic estrogen ethinylestradiol (Kuhl, 2005; Evans and Sutton, 2015). Moreover, estradiol is also used in therapies to treat prostate and breast cancer (Ali Shah, 2015; Coelingh Bennink et al., 2017). In 2016, more than 13 million prescriptions of estradiol were made allowing for the 59th most prescribed medication in the United States. In this scenario, there is therefore an urgent need to sensitively and precisely detect estradiol in a cost-effective and easy way (The top 300 of 2019, 2018). The development of ePAD for hormones detection can meet the requirements of novel challenging (bio)analytical studies, improving the quality of life, providing reliable qualitative results, as well as reducing the time and cost of analysis in comparison with conventional assay procedures (e.g., ELISA and chromatography) (Şimşek et al., 2015; Lim et al., 2017).

**FIGURE 3:**
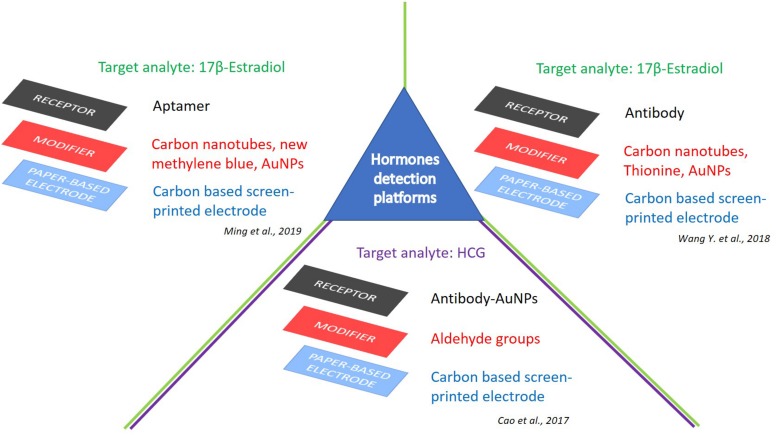
e-PAD platforms for detection of hormones. Target analytes: 17β-estradiol (Wang Y. et al., 2018; Ming et al., 2019), and HCG (Cao et al., 2017).

Wang Y. et al. (2018) recently proposed a label-free integrated ePAD for the highly sensitive electrochemical detection of 17β-E2. The authors fabricated by wax printing a three electrode microfluidic device, then modified with a multi-walled carbon nanotubes/thionine/gold nanoparticles composite, directly synthesized on the working electrode, for the immobilization of anti-E2 (Figure 3). The resulted device detected 17β-E2 as low as 10 pg mL^–1^, with a linear range from 0.01 to 100 ng mL^–1^. The selectivity of the immunoassay was evaluated in the presence of follicle-stimulating hormone, luteinizing hormone, glutamic acid, ascorbic acid, uric acid, neuron-specific enolase, and carcinoembryonic antigen, which provided a variation of peak currents of 3.62, 1.77, 6.33, 4.93, 0.81, 7.57, and 5.79%, respectively. Finally, clinical serum samples were also analyzed with high sensitivity and good accuracy, with a relative deviation of 8.10–15.40% from standard methodologies (i.e., a commercial-available electrochemiluminescence apparatus from Roche).

Concerning this topic, a noteworthy contribution was reached by Ming et al. (2019) with the development of a folding aptasensor platform equipped of microfluidic system to obtain a label-free electrochemical detection of 17β-E2. This device encompasses filter holes, reaction chambers, microfluidic channels, and three-electrode system, as well. The detection sensitivity and aptamer immobilization was enhanced by exploiting a novel nanoassembly, consisting of amine-functionalized single-walled carbon nanotubes/new methylene blue/gold nanoparticles to modify the working electrode (Figure 3). A detection limit of 5 pg mL^–1^ (S/N = 3) was observed with a linear range comprised between 10 pg mL^–1^ and 500 ng mL^–1^ (*R*^2^ = 0.993) (Ming et al., 2019).

Following the growing trend of infertility, the clinical use of fertility-related hormones has increased in recent years, and the intake of gonadotropin, beneficial for ovarian stimulation, is part of this medical record (Leão and Esteves, 2014; Murray et al., 2018; Lunenfeld et al., 2019). Moreover, the chorionic gonadotropin (HCG) is a well-known and important biomarker present in the blood and urine of pregnant women, with pivotal roles against complications during prenatal care; furthermore, elevated levels of HCG were found in many cancerous tumors. For these reasons, HCG sensing in human urine or serum is highly sought after (Theofanakis et al., 2017). Recently, Cao et al. (2017) proposed an electrochemical detection for HCG in a linear range from 1 mIU mL^–1^ to 100 IU mL^–1^, with a detection limit of 0.36 mIU mL^–1^. The authors fabricated hydrophilic test zones on an aldehyde-functionalized screen-printed electrode functionalized with capture antibodies (Ab1) (Figure 3). Then, primary signal antibody functionalized gold nanoparticles (GNPs/Ab2) and alkaline phosphatase conjugated secondary antibody (ALP-IgG) were used for the detection of HCG by differential pulse voltammetry. This disposable, efficient, sensitive, and low-cost ePAD was tested on real human serum, showing a great potential for the development of point-of-care devices (Cao et al., 2017).

### Anti-inflammatory Drugs

Non-steroidal anti-inflammatory drugs (NSAIDs) represent another class highly consumed over the world as over-the-counter products. These compounds can face inflammation and swelling states, and these actions are often associated with a painkiller effect. Unfortunately, NSAIDs are also known for the side effects that can determine in patients who abuse or use them for long time (Brune and Patrignani, 2015; Aguilar-Lira et al., 2017).

The API Diclofenac sodium (sodium [*o*-(2,6-dichloroanilino) phenyl] acetate) belongs to the NSAID class, prescribed as anti-pyretic, anti-rheumatic, analgesic, anti-inflammatory in case of degenerative disease, arthritis, musculoskeletal injuries, ankylosing spondylitis, post-surgery analgesia, osteoarthritis, and also in sport injuries. Being its quantification of great importance for the clinical (e.g., quality and therapeutic control) and environmental (e.g., emerging contaminant determination) sectors, there is a huge commercial demand for sensitive, selective, accurate, and cost-effective tools for diclofenac sodium detection devoted to pharmaceuticals, medical and biomedical sectors (Shalauddin et al., 2019).

**FIGURE 4:**
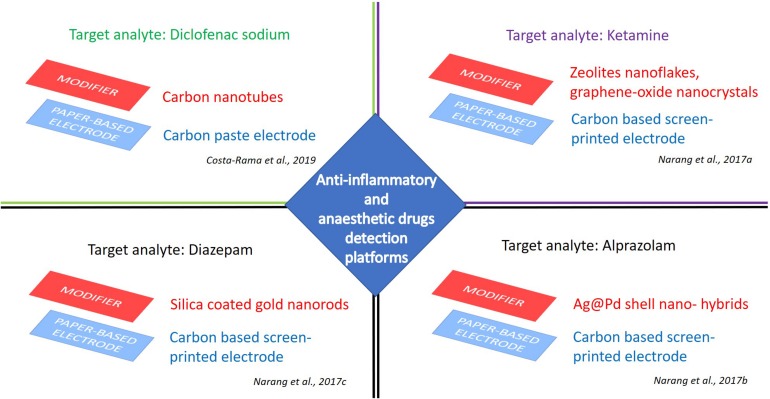
e-PAD platforms for detection of anti-inflammatory and anesthetic drugs. Target analytes: Diclofenac sodium (Costa-Rama et al., 2019), ketamine (Narang et al., 2017a), alprazolam (Narang et al., 2017b), and diazepam (Narang et al., 2017c).

Costa-Rama et al. (2019) reported a smart ePAD for diclofenac detection, reaching a very competitive limit of detection (70 nM) with an RSD of about 5%, exploiting the possibility of sample pre-concentration (Costa-Rama et al., 2019). The easy-to-handle device was fabricated using a carbon-ink paper-based working electrode and two metallic wires, provided by a gold-plated standard connector, as reference and counter electrodes (Figure 4). This multiplex configuration enabled sample pre-concentration, sample, and detection, providing a wide dynamic range between 0.10 and 100 μM, with two linear concentration ranges (i.e., 0.10–5.0 μM and 5.0–100 μM).

### Anesthetic Drugs

Several APIs are daily used in medical practice to induce anesthesia, which means a temporary loss of sensation or awareness. Among them, ketamine is an important anesthetic drug widely administered in medication to anesthetize patients, as depression remedy and for post-operative pain management.

Narang et al. (2017a) explored the possibility to develop an ultrasensitive technique for ketamine electro-sensing by a nano-hybrid micro fluidic ePAD device. A paper chip modified with nanocrystals of zeolites and graphene oxide nanoflakes was designed for ketamine detection by cyclic voltammetric technique (Figure 4). The accuracy of this ePAD was evaluated by measuring ketamine in alcoholic and non-alcoholic drinks. This configuration can represent a positive perspective for pharmaceutical purposes, as well as furnish advantages over conventional three electrode systems, as these are easy to prepare, economical, portable and disposable after use. Benzodiazepine group is a significant segment in anesthetic drugs, generally prescribed as psycho-pharmaceuticals (e.g., for anxiety, tension, insomnia) (Murphy et al., 2016). In recent years, their abusing in combination with opioids and alcohol caused many emergency department visits and related deaths (Schmitz, 2016). Alprazolam is one of the benzodiazepine anti-depressive therapeutically administered as anxiolytics, tranquilizers, and muscle relaxants (Tan et al., 2011). Its overdose determines acute drowsiness, muscle numbness, and coordination problems. Thus, alprazolam monitoring is of utmost importance to clinicians and forensic toxicologists (Donoghue and Lader, 2010). The conventional and time-consuming methods exploited for the analysis of alprazolam in biological fluids and pharmaceutical formulation today can be coupled and/or replaced with ePAD technology, with a first attempt achieved with a facile lab-paper chip based on urchin like Ag@ Pd shell nano-hybrids (Figure 4). The alprazolam detection was performed in a reliable and fast manner by cyclic voltammetric technique in buffer solutions at clinically relevant concentrations (detection limit of 0.025 ng/l and linear range 1–300 ng/mL), as well as in real time samples, as urine and serum, with good correlation (99%) (Narang et al., 2017b).

An innovative device based on ePAD technology was developed also for the detection of diazepam, a sedative, anxiety-relieving, and muscle-relaxing API belonging to the benzodiazepine category. For this purpose, silica coated gold nanorods (Si@GNRs) were synthesized and drop cast on an electrochemical microfluidic paper-based device (Figure 4). The modified paper-based electrode showed a stable cyclic voltammetric response in an analyte concentration range from 3.5 nM to 3.5 mM. This device was tested for diazepam in spiked human urine samples, but the authors recommended this configuration also for the determination of serum metabolites (Narang et al., 2017c). All these qualities, in terms of response speed, sample volume required and signal reliability, could rightly motivate the choice of ePAD technology rather than the conventional methods used for the determination of alprazolam in biological fluids and pharmaceutical formulation (e.g., UV spectroscopy, HPLC, GC-MS, and LC-MS) (Madea and Mußhoff, 2009).

### Stimulants of the Central Nervous System

Stimulants are natural and synthetic APIs affecting the central nervous system (CNS) activity (e.g., caffeine, cocaine, amphetamines, and methamphetamine). Their effect can be useful for the treatment of prolonged fatigue, inability to concentrate, excessive sleepiness, attention deficit disorder as well as prolonged depression. The CNS stimulants can differ in the level of neurotransmitters produced and in their time of action. In this contest, the chemical research is very dynamic, and enhanced products have been already achieved as in the case of methamphetamine, which is obtained adding a methyl group to amphetamine molecule. This improvement determined a longer drug effect, and a better penetration into the brain in comparison with amphetamine, as well as less detrimental consequences on heart (Hegadoren et al., 1999; Martinez-Price et al., 2002). However, these APIs are frequently abused beyond medical and therapeutic context, including athletes and recreational users, causing serious public health concern (Napper et al., 2010).

**FIGURE 5:**
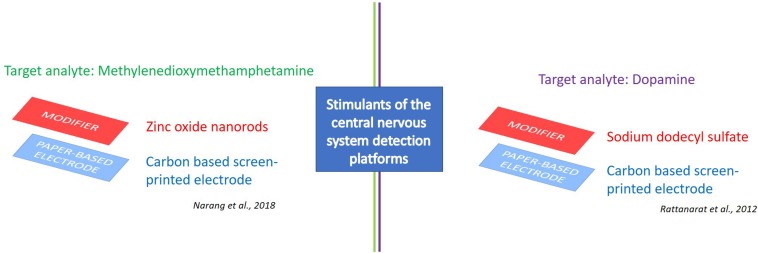
e-PAD platforms for detection of stimulants of the central nervous system. Target analytes: Methylenedioxymethamphetamine (Narang et al., 2018), and dopamine (Rattanarat et al., 2012).

The ePAD technology aims to find smart solutions for the urgent need to detect these APIs for both pharmaceutical and forensic diagnostic applications. Intriguing scientific results are already available, such as the ePAD for the drug methylenedioxymethamphetamine. This device was equipped with a working electrode modified with zinc oxide nanorods (ZnONRs) (Figure 5), showing optimum response in cyclic voltammetry at 7.0 pH with a detection limit of 0.1 μM and linear range of 1 μM–1 mM. Satisfactory results in terms of recovery were also reported during the evaluation test of the sensor (Narang et al., 2018). As stated by the authors, the proposed ePAD shows many advantageous features of being simple, low-cost, consistent and disposable. This allows for cost-effective, simple and fast analysis also in the field for pharmaceutical applications, thus avoiding expensive samples transportation to the laboratories as well as complex and long-lasting analytical procedures.

Another noteworthy example is the ePAD designed by Rattanarat et al. (2012) for the selective determination of dopamine using square-wave voltammetry, with a detection limit of 0.37 μM and a linear range of 1–100 μM. The device was constituted by: (i) a top layer of SU-8 photoresist defining a hydrophilic sample application spot on the filter paper; (ii) a middle layer made of a transparency film containing two holes for sample pre-concentration and surfactant; and (iii) a screen-printed carbon electrode forming the bottom layer for the electrochemical measurements (Figure 5). Prime measurements were obtained when the paper was modified with the anionic surfactant sodium dodecyl sulfate, with a current increase of five-fold and an advanced selectivity of the device. On the contrary, the use of non-ionic Tween-20 had no effect and cationic tetradecyltrimethylammonium bromide surfactants showed reduced current signals, respectively, highlighting the capability of paper to both store the reagents and increase the analytical response.

## Conclusion and Future Remarks

Pharmaceutical industry requests a reorganization of drug production system, which is excessively long and expensive. This remarkable revolution could praise the ePAD technology, which, despite not yet fully exploited, show relevant features in terms of sustainability, sensitivity, robustness, and repeatability. In this review, the reliability of ePADs for pharmaceutical field was reported as highlighted in Table 3, and the pivotal role of the interdisciplinary convergence of microfluidics and nanotechnology, as well as the engagement of sustainable materials, was described as a key motif of ePAD rapid improvement. Moreover, the representative scientific goals obtained and the multiple applications of ePAD technology were described in pre-clinical and early clinical phases of drug development, pharmaceutical products quality assessment. Furthermore, the rational of this detailed description on ePADs aims to provide new fields of action of these interesting devices, such as in counterfeiting and illicit drug screening. In particular, the exploitation of ePADs can be extremely positive for the detection of adulterants (e.g., amphetamines and paracetamol), often not considered in clinical or forensic toxicology, but clue of poor manufacturing techniques, and with adverse health consequences (Cole et al., 2011). Several scientific evidences suggest that illicit drugs are commonly adulterated with benign substances, as well as those that enhance or mimic the effects, and others to facilitate the administration. For these reasons, novel approach of investigation and monitoring, such as ePADs, are strongly required to furnish the state of art concerning the adulteration practices and appropriate countermeasures.

**TABLE 3:** Snapshot on innovative and representative ePADs for the pharmaceutical sector.

**Type of ePADs**	**Type of paper**	**Working electrode**	**Active reagent**	**Linear range**	**LOD**	**Sample**	**Comments**	**Advantages**	**References**
Paper-based screen-printed carbon electrode	Filter	Inkjet-printed PANI modified electrode	Ascorbic acid	From 30 to 270 μM	30 ± 3 μM	Acetate buffer	Measurements carried out by using chronoamperometry	High-performance, low-cost and disposability	Kit-Anan et al. (2012)
Paper-based screen-printed sensor produced by a CO_2_ laser etching system	Whatman #1	Carbon paste microelectrode	Cysteine	From 100 to 500 μM	4.8 μM	Acetate buffer	Measurements carried out by using linear sweep voltammetry and cobalt phthalocyanine as a redox mediator	Fast response time is achieved due to the low electric double layer capacitance. This property allows rapid changes in the concentration of redox-active species to be monitored at nanosecond time scales	Santhiago et al. (2013)
SDS-modified paper-based screen-printed analytical device	Filter	Carbon paste electrode	Dopamine	From 1 to 100 μM	0.37 μM	Human serum	Measurements carried out by using square-wave voltammetry	Low cost, easy-to-use, portable paper-based device	Rattanarat et al. (2012)
Hydrothermally synthesized zinc oxide nanorods incorporated on a paper-based screen printed device	−	Carbon paste electrode coated with zinc oxide nanorods	Methylenedi- oxymethamphe- tamine	From 1 μM to 1 mM	0.1 μM	Biological fluids	Measurements carried out by using cyclic voltammetry	The reported EPAD exhibits sensitive, highly specific and fast response toward MDMA in both aqueous solution and spiked bio- logical sample	Narang et al. (2018)
Lab on paper chip integrated with silica coated gold nanorods	−	Carbon paste electrode modified with Si@GNRs	Diazepam	From 3.5 nM to 3.5 mM	1.5 nM	Human urine	Measurements carried out by using cyclic voltammetry	Compared to silicon, glass and metal devices, μPAD offers advantageous features such as cost-effective, non-requirement of complex fabrication processes. The integrated with Si@GNRs provides better results in terms of response limit.	Narang et al. (2017c)
Paper-based device integrated with urchin like Ag@ Pd shell nano-hybrids	Whatman #1	Carbon paste electrode modified with MB doped silver core shell palladium nano-hybrids	Alprazolam	From 1 to 300 ng/mL	0.025 ng/l	Phosphate buffer solution	Measurements carried out by using cyclic voltammetry using MB as redox transition substance	The nano-hybrid array based μPAD is really quick: 10 s of incubation time	Narang et al. (2017b)
Carbon black nanomodified inkjet-printed sensor	P_e:smart^®^	Carbon paste electrode coated with carbon black N 220	Ascorbic acid	From 0 to 5 mM	0.15 mM	Dietary supplement	Measurements carried out by using cyclic voltammetry	An integrated printed paper-based electrochemical device using a single production process with advantages in terms of cost and time of production.	Cinti et al. (2018)
Microfluidic paper based device integrated with nano zeolite–graphene oxide nanoflakes	Whatman #1	Carbon paste electrode modified with nanocrystal of Zeo–GO	Ketamine	From 0.001 to 5 nM/m	0.001 nM/m	Alcoholic and non-alcoholic drinks	Measurements carried out by using cyclic voltammetry	This paper-based chip is much cheaper than metal electrodes-based devices and less response time (2 s)	Narang et al. (2017a)
Multiplexed paper-based electrochemical device	Whatman #1	Carbon paste electrode modified with MWCNTs and CNF	Diclofenac	From 0.10 to 5 μM and from 5 to 100 μM	70 nM	Standard solution	Measurements carried out by using linear sweep voltammetry	This multiplexed paper-based platform performs several preconcentration step and measurements simultaneously reducing the analysis time	Costa-Rama et al. (2019)
Electrochemical immunofiltration analysis incorporated in a paper-based screen-printed microfluidic devices	Whatman #1	Carbon paste electrode modified with	Human chorionic gonadotropin	From 1.0 mIU/mL to 100.0 IU/mL	0.36 mIU/mL	Standard solution	Measurements carried out by using differential pulse voltammetry and a sensitive immune detection method by using ALP-IgG labeled immunogold as the signal probe	The paper-based immunofiltration analysis exhibits advantages including low-cost, simple operation time (15 min of incubation time) and sample-saving without professional conditions	Cao et al. (2017)
A microfluidic paper-based electrochemical aptasensor	Whatman #1	Carbon paste electrode modified with NH2- SWCNTs/NMB/AuNPs nanocomposite	17β-Estradiol	From 10 pg/mL to 500 ng/mL	5 pg/mL	Standard solution	Measurements carried out by using differential pulse voltammetry	Fast analysis time: a reaction time of 60 min allowed Estradiol and the aptamer to sufficiently bind.	Ming et al. (2019)
Paper-based device composed of two electrochemical cell compartment and an array of 4-working electrodes	Filter	Carbon paste electrode	Ascorbic acid	From 2.6 to 28.7 mM	0.40 mM	Standard solution	Measurements carried out by using square wave voltammetry	The ePAD was fabricated by a rapid prototyping process using an inexpensive home cutter printer (∼US$ 300). An array of four working electrode for each electrochemical cell allow simultaneous multi analysis.	de Oliveira et al. (2019)
Label-free integrated screen-printed paper-based immunosensor	Filter paper	Carbon paste electrode modified with MWCNTs/THI/AuNPs nanocomposite	17β-Estradiol	From 0.01 to 100 ng/mL	10 pg/mL	Standard solution	Measurements carried out by using differential pulse voltammetry and THI as electrochemical mediator	This label-free device allows for rapid and user friendly test (incubation time for the binding of antigen and corresponding antibody last for about 15 min). Low-cost: the expense of a sample test was about $ 0.3.	Wang Y. et al. (2018)

*ePADs, electrochemical paper-based analytical devices; PANI, polyaniline; MB, methylene blue; Ag, silver; Pd, palladium; Si@GNRs, silica coated gold nanorods; Zeo, zeolite; GO, graphene oxide; RE, pseudoreference; CE, counter; MWCNTs, multi-walled carbon nanotubes; CNF, carbon nanofibers. ALP-IgG, alkaline phosphatase conjugated secondary antibody (immunoglobulin G); NH2-SWCNTs, amine-functionalized single-walled carbon nanotubes; NMB, new methylene blue; AuNPs, gold nanoparticles. THI, thionine.*

However, ePAD technology for pharmaceutical compounds is struggles to reach the market, and this is probably due to some technical limitations not yet overcome, such as sensitivity and reproducibility, taking into account that the repeatability is often at the level of RSD 10%, because the development of ePAD is still at research level. Indeed, a future industrialization is capable to boost the fabrication of sensors with increased reproducibility with lower RSD% values, exploiting the industrial fabrication processes (e.g., use of BioDot, low volume precision dispensing equipment, for nanomaterial modification of working electrode). To face this drawback, strategies for signal enhancement need to be evaluated to measure traces of pharmaceutically active compounds. For example, metal nanoparticles and carbon materials demonstrated their benefits for more sensitive ePADs. Long-term stability and reproducibility of such devices should be also taken into consideration for marketable purposes, as well as the requirement for micro- and nano-volumes of analysis to provide minimized invasiveness for the patients in case of pharmacokinetic bioanalysis.

Moreover, the main challenge relies on the admission of ePADs as accurate and reliable analytical tools in quality tests and assurance purposes carried out at pharmaceutical industries by government agencies, actually hindering ePAD application. The requirement of certified quality tests probably restricts the development of ePADs for pharmaceutical compounds, actually limited to few devices, when compared with the huge number of ePADs developed for the detection of biomarkers in biological fluids and pollutants in environmental samples (Mettakoonpitak et al., 2016; Arduini et al., 2017). Considering the overall scenario, further research efforts should be devoted toward the development of novel ePADs for the detection of emerging APIs as well to the validation of developed ePADs to better establish their reliability and effectiveness.

## Author Contributions

VS, AA, VC, VM, and LF involved in the writing of the manuscript. FA and DM involved in the supervision of the manuscript.

## Conflict of Interest

FA is involved in the company SENSE4MED. The remaining authors declare that the research was conducted in the absence of any commercial or financial relationships that could be construed as a potential conflict of interest.
